# Fluorogenic Substrates for Visualizing Acidic Organelle Enzyme Activities

**DOI:** 10.1371/journal.pone.0156312

**Published:** 2016-05-26

**Authors:** Fiona Karen Harlan, Jason Scott Lusk, Breanna Michelle Mohr, Anthony Peter Guzikowski, Robert Hardy Batchelor, Ying Jiang, John Joseph Naleway

**Affiliations:** 1 Research and Development, Marker Gene Technologies, Inc., Eugene, OR, United States of America; 2 Sonas BioPharma, Inc., Eugene, OR, United States of America; University Hospital S. Maria della Misericordia, Udine, ITALY

## Abstract

Lysosomes are acidic cytoplasmic organelles that are present in all nucleated mammalian cells and are involved in a variety of cellular processes including repair of the plasma membrane, defense against pathogens, cholesterol homeostasis, bone remodeling, metabolism, apoptosis and cell signaling. Defects in lysosomal enzyme activity have been associated with a variety of neurological diseases including Parkinson’s Disease, Lysosomal Storage Diseases, Alzheimer's disease and Huntington's disease. Fluorogenic lysosomal staining probes were synthesized for labeling lysosomes and other acidic organelles in a live-cell format and were shown to be capable of monitoring lysosomal metabolic activity. The new targeted substrates were prepared from fluorescent dyes having a low pKa value for optimum fluorescence at the lower physiological pH found in lysosomes. They were modified to contain targeting groups to direct their accumulation in lysosomes as well as enzyme-cleavable functions for monitoring specific enzyme activities using a live-cell staining format. Application to the staining of cells derived from blood and skin samples of patients with Metachromatic Leukodystrophy, Krabbe and Gaucher Diseases as well as healthy human fibroblast and leukocyte control cells exhibited localization to the lysosome when compared with known lysosomal stain LysoTracker^®^ Red DND-99 as well as with anti-LAMP1 Antibody staining. When cell metabolism was inhibited with chloroquine, staining with an esterase substrate was reduced, demonstrating that the substrates can be used to measure cell metabolism. When applied to diseased cells, the intensity of staining was reflective of lysosomal enzyme levels found in diseased cells. Substrates specific to the enzyme deficiencies in Gaucher or Krabbe disease patient cell lines exhibited reduced staining compared to that in non-diseased cells. The new lysosome-targeted fluorogenic substrates should be useful for research, diagnostics and monitoring the effect of secondary therapeutic agents on lysosomal enzyme activity in drug development for the lysosomal storage disorders and allied diseases.

## Introduction

Lysosomes are acidic cytoplasmic organelles that are present in all nucleated mammalian cells. Lysosomes have been found to be involved in a variety of cellular processes including repair of the plasma membrane, defense against pathogens, cholesterol homeostasis, bone remodeling, metabolism, apoptosis and cell signaling. To date, more than 50 acidic hydrolytic enzymes have been identified that are involved in ordered lysosomal degradation of proteins, lipids, carbohydrates and nucleic acids. Functional deficiencies in these lysosomal enzymes are indicative of a number of disease states. Lysosomes are also involved in metabolism and catabolism of foreign molecules that are brought into the cell by endocytosis, acting as a first line of defense against foreign bacterial or viral infection. The acidic pH of lysosomes is critical to the process by which lipid-enveloped viruses enter the cytoplasm after their cellular uptake by receptor-mediated endocytosis. Acidic organelles have also been shown to be responsible for digestion of high molecular weight proteins, oligosaccharides, glycolipids or peptides by the cell. In addition, they are often involved in therapeutic drug metabolism.

The lysosomal storage diseases are a family of genetic human metabolic diseases that, in their severest forms, cause mortality due to a variety of conditions such as progressive neurodegeneration, organ failure or cardiac arrest. They are caused by mutations in the genes encoding lysosomal glycohydrolases that catabolize glycosphingolipids within the lysosome, activator proteins or integral membrane proteins. When there is a lysosomal enzyme deficiency, the deficient enzyme's undegraded substrates gradually accumulate within the lysosomes causing a progressive increase in the size and number of these organelles within the cell. This accumulation within the cell eventually leads to malfunction of the organ and to the symptoms of a lysosomal storage disease, with these symptoms depending on the particular enzyme deficiency. More than fifty distinct, inherited lysosomal storage diseases have been characterized in humans.

Gaucher disease, the most common lysosomal storage disease in humans, is caused by a deficiency in the lysosomal enzyme glucocerebrosidase (hGCB; GBA1; glucosylceramidase; acid β-glucosidase; EC 3.2.1.45). This deficiency leads to an accumulation of the enzyme's substrate, glucocerebroside in cells and tissues, leading to anemia, bone deterioration, and in the case of type II and III seizures and brain damage. Recent developments have also indicated a probable link between Gaucher disease and Parkinson’s disease where both carriers of a GBA1 mutation and disease sufferers show earlier onset and more severe symptoms of Parkinson’s. Krabbe disease (also known as globoid cell leukodystrophy or galactosylceramide lipidosis) is a rare, often fatal degenerative lysosomal disorder that causes storage of unmetabolized lipids that affect growth of the nerve's protective myelin sheath. It is caused by a deficiency in galactosylceramidase (GALC; ED 3.2.1.46). New, sensitive and specific assays for monitoring such lysosomal enzyme activities in living cells will be of significant value in monitoring the success of current therapies and for discovery of new therapeutic strategies for diseases of lysosomal origin.

Weakly basic amines have been shown to selectively accumulate in cellular compartments with low internal pH. When further linked to chromophores or fluorescent probes, they can be used to label these compartments. Among these is the frequently used acidotropic probe, N-(3-((2,4-dinitrophenyl)amino)propyl)-N-(3-aminopropyl)methylamine, dihydrochloride (referred to as DAMP). DAMP is not fluorescent and requires fixation and permeabilization of the cell, followed by the use of anti-DNP antibodies conjugated to a fluorophore, an enzyme or ferritin in order to visualize the staining pattern. The fluorescent probes neutral red and acridine orange are also commonly used for staining acidic organelles. In addition, fluorescently labeled latex beads and macromolecules, such as FITC-dextran, have been used to label lysosomes or record lysosomal pH, but they lack specificity and are not well retained in the organelles, particularly after fixing and permeabilization. Dansyl cadaverine has been described as a lysosomotropic reagent [1,2]. But recent reports also describe it as a useful label for autophagic vacuoles, as it fails to label either endosomal compartments or lysosomes [3]. Perfluorobenzylamides and chloromethyl derivatives of fluorophores have also been utilized for monitoring lysosomal metabolism in live cells, but their staining patterns are often non-specific. Finally, certain dipyrrometheneboron difluoride (BODIPY) fluorophores linked to a weak base that is only partially protonated at neutral pH have been used for general labeling of lysosomes [4]. However as none of these probes have shown utility for monitoring specific enzyme activities within lysosomes, they are not useful for analysis of enzyme defects resulting in pathogenic conditions or of the effect of secondary drug administration on lysosomal enzyme activities in live cells.

Of the numerous lysosomal storage assay diagnostic systems that have been reported, the majority utilize either fluorescent (4-methylumbelliferyl) substrates, chromogenic (nitrophenolic glycosides), glycolipids labeled with fluorescent dyes or radioactive substrates for detection of lysosomal glycosidase activities. These methods, however, utilize either cell lysate from cells or tissue homogenates, HPLC or TLC separation of enzymatic products and UV or fluorescent analysis or other complex analysis techniques that are not transferable to a live cell format. None of these assays, therefore, are well designed for live-cell high-content systems detection, and require either cell lysis or extensive cell preparation steps. In addition, the fluorescent dyes used in these assays are often not amenable to the low pH environment of the lysosome and therefore do not permit imaging in the lysosome in its native environment.

**Fig 1:**
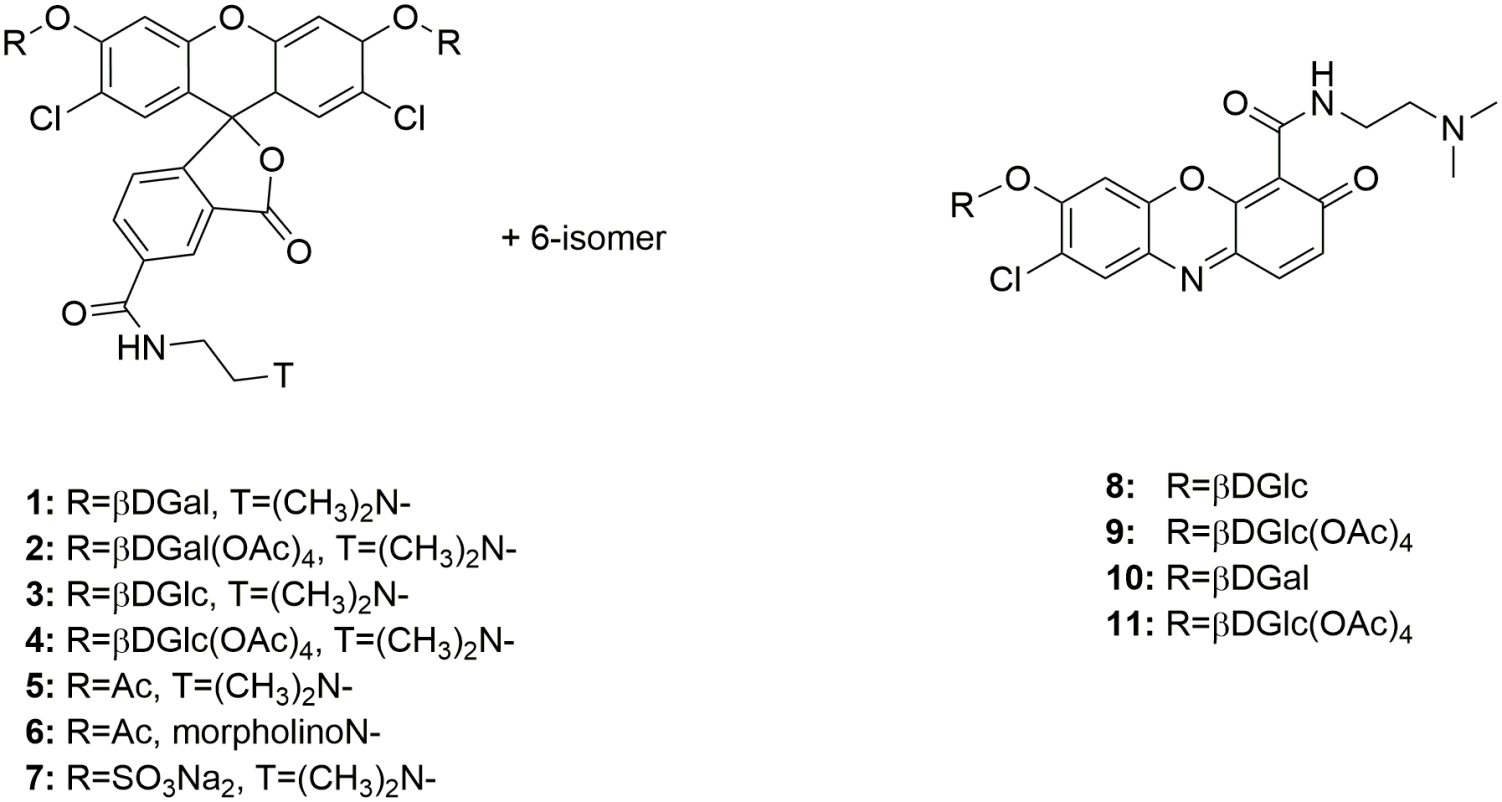
Structures of Lysosome Targeted Fluorogenic Enzyme Substrates.

We herein describe a series of fluorogenic enzyme substrates (Fig 1) derived from fluorophores with low pKa values that show utility for labeling and tracing enzyme activity within acidic organelles, and in particular in lysosomes, within living cells. These new probes contain weakly basic amine targeting groups that allow them to selectively accumulate in acidic organelles with low internal pH and show promise for use in investigating the specific enzyme levels responsible for biosynthesis, degradation and recycling of cellular components and for measuring specific enzyme defects involved in a number of human diseases associated with abnormal enzyme activity in lysosomes within live cells.

## Materials and Methods

### Cell Culture Methods

Human skin fibroblasts from a healthy specimen as well as from patients with Gaucher disease type I, II and III were obtained from the Istituto Giannina Gaslini (Genova, Italy)[5] and the Coriell Institute for Medical Research (Camden, New Jersey). Cells were maintained in Eagle’s Minimum Essential Medium (EMEM) (Hyclone) supplemented with 10% Fetal Bovine Serum (Atlanta Biologicals) and 1X Pen/Strep (Gibco). Cells were grown to 90% confluence and passaged by splitting at a 1:5 ratio. Cells were incubated at 37°C, with 5% CO_2_ atmosphere.

Immortalized B-Lymphoblast cells from both healthy individuals of different ages and genders, Gaucher Disease Patients and Krabbe Disease Patients were obtained from the Coriell Institute for Medical Research (Camden, New Jersey). Cells were maintained in RPMI 1640 Medium (HyClone) supplemented with 15–20% Fetal Bovine Serum (Atlanta Biologicals) and 1X Pen/Strep (Gibco). Cells were maintained at a density between 2x10^5^ and 5x10^5^ at 37°C, with 5% CO_2_ atmosphere.

The cell lines used in this study were as follows: AG06173 Normal Human Fibroblasts; GM00372 Gaucher I Human Fibroblasts; GM04394 Gaucher I Human Fibroblasts; GM02627 Gaucher II Human Fibroblasts; GM08760 Gaucher II Human Fibroblasts; F0971986 Gaucher III Human Fibroblasts; GM06805 Krabbe Human B-Lymphocytes; GM14643 Normal Human B-Lymphocytes; and GM10870 Gaucher I Human B-Lymphocytes.

### Staining of Lysosomes with Fluorogenic Substrates

The desired substrate was dissolved in DMSO to prepare a 10mM stock solution. This stock solution was kept sealed in small aliquots at -20°C until use with exposure to light minimized and thawed completely at room temperature prior to use. Labeling solutions were prepared by adding substrate stock solution to fresh serum-free culture medium (37°C) in amounts sufficient to make final substrate concentrations ranging from 1–200 μM, with the final concentration of DMSO not exceeding 2%.

The labeling solution containing the prepared substrate was added to cells grown in 96-well black with optical bottom microplates (Nunc-Thermo Scientific 165305), followed by incubation at 37°C in 5% CO_2_ atmosphere for 1 to 16 hours. The cells were then washed with pre-warmed PBS followed by addition of Opti-Klear^™^ Live Cell Imaging Buffer (Marker Gene Technologies, Inc., Eugene, OR) containing 10ug/ul Hoechst 33342 (Sigma-Aldrich)(100 uL per well). Cells could also be imaged after addition of PBS, fresh FCS free media or other imaging buffers prior to viewing. Cells were examined under a fluorescence microscope equipped with appropriate filter sets, e.g. FITC and DAPI.

### Determination of Substrate Localization by Co-Staining with Anti-LAMP1 Antibody

Live fibroblasts derived from a Gaucher III patient (Gaslini Institute cell line F0971986) grown in 96-well black glass bottomed microplates (In Vitro Scientific, Mountain View CA) were incubated with 5uM 5(6)-(2-dimethylaminoethyl)carboxamido-2',7'-dichlorofluorescein diacetate (**5**) for 1 hour at 37°C/5% CO_2_. Staining media was then removed from the cells and 100uL of fixative (4% formaldehyde/1% glutaraldehyde in PBS) added, followed by incubation at room temperature for 15 minutes with shaking. Cells were then washed 3 times with PBS and then permeabilized with 100uL of 0.2% Triton-X in PBS for 5 minutes at room temperature. Cells were washed 3 times in blocking buffer (1% BSA in PBS) and then incubated overnight at 4°C in blocking buffer. Primary antibody, rabbit anti-LAMP1 pAb (Abcam, Cambridge, MA) was then applied at 1.6ug/mL in blocking buffer and the cells incubated at room temperature for 1 hour. Cells were washed 3 times in blocking buffer. Secondary antibody, Alexa Fluor^®^ 555 conjugated Goat Anti-Rabbit (Abcam, Cambridge MA) was added at a 1:500 dilution in blocking buffer and incubated for 1 hour at room temperature protected from light. Cells were washed 3 times in PBS with Hoechst 33342 (Sigma Aldrich) added to the second wash. Images were then captured on a Zeiss Axio Observer A1 inverted microscope fitted with a 40X lens and DAPI, FITC and TAMRA filter sets.

### Determination of Substrate Localization by Co-Staining with LysoTracker^®^ Red DND-99

Live human fibroblasts derived from a healthy individual (TIG-1 cell line Coriell Cell Repository catalog number AG06173) were incubated with 5uM 5(6)-(2-dimethylaminoethyl)carboxamido-2',7'-dichlorofluorescein-3’,6’-di-O-β-D-glucopyranoside, octaacetate (**4**) for 16 hours then stained with 75nM LysoTracker^®^ Red DND-99 (Life Technologies, Carlsbad CA) for 30 minutes at 37°C in 5% CO_2_. Cells were then washed 3 times in PBS and imaged in Opti-Klear^™^ Live Cell Imaging Buffer (Marker Gene Technologies, Inc. Eugene, OR) containing 10ug/mL Hoechst 33342 using an AMG EVOS Auto FL microscope using DAPI, GFP and TxRed lightcubes, Cells could also be bathed in PBS, fresh FCS-free media or other imaging buffers prior to viewing.

### Reduction in Lysosomal Metabolic Activity by Chloroquine Inhibition

Healthy donor fibroblasts (TIG-1 cell line Coriell Cell Repository catalog number AG06173) plated in 96 well glass bottomed plates (In Vitro Scientific, Mountain View CA) were incubated for 16 hours with or without 300uM chloroquine at 37°C/5% CO_2_. The inhibitor containing media was removed and replaced with serum free media containing 10uM 5(6)-dimethylaminoethylcarboxamido-2',7'-dichlorofluorescein diacetate (**5**) and cells were incubated for 2 hours at 37°C/5% CO_2_. Staining media was removed and cells were washed 3 times with PBS prior to the addition of Opti-Klear^™^ Live Cell Imaging Buffer (Marker Gene Technologies, Inc. Eugene, OR) for imaging. Cells could also be bathed in PBS, fresh FCS-free media or other imaging buffers prior to viewing. Images were captured on a Zeiss Axio Observer A1 upright microscope fitted with a 40X lens and FITC filter set.

### Measurement of Glucocerebrosidase Activity after Conduritol β- Epoxide Inhibition

Healthy donor fibroblasts (TIG-1 cell line Coriell Cell Repository catalog number AG06173) were plated in 96 well black with optical bottom microplates (Nunc-Thermo Scientific) and incubated for 16 hours with varying concentrations of Conduritol B Epoxide (CBE, Toronto Research Chemicals, Toronto ON) (5–50 uM) as well as a no CBE control, then treated with 5uM 5(6)-(2-dimethylaminoethyl)carboxamido-2',7'-dichlorofluorescein-3’,6’-di-O-β-D-glucopyranoside, octaacetate (**4**) at 37°C/5% CO_2_ overnight. The media containing the β-glucosidase substrate and inhibitor was removed and cells were washed 3 times with PBS prior to the addition of Opti-Klear^™^ Live Cell Imaging Buffer (Marker Gene Technologies, Inc. Eugene, OR) containing 10ug/ml Hoechst 33342. Cells could also be bathed in PBS, fresh FCS-free media or other imaging buffers prior to viewing and images were captured using an AMG EVOS Auto FL with a 40X objective and GFP and DAPI lightcubes.

### Requirement of Acetate Groups for Live Cell Permeability

Healthy donor fibroblasts (TIG-1 cell line Coriell Cell Repository catalog number AG06173) plated into 96-well black with optical bottom microplates (Nunc-Thermo Scientific) were incubated for 16 hours with 5(6)-(2-dimethylaminoethyl)carboxamido)-2’7’-dichlorofluorescein-3’,6’-di-O-β-D-glucopyranoside (**3**) or 5(6)-(2-dimethylaminoethyl)carboxamido-2',7'-dichlorofluorescein-3’,6’-di-O-β-D-glucopyranoside octaacetate(**4**), at 12.5uM and 25uM. Prior to imaging, staining media was removed and cells washed 3 times with PBS. Cells were then bathed in Opti-Klear^™^ Live Cell Imaging Buffer (Marker Gene Technologies, Inc. Eugene, OR) containing 10ug/ml Hoechst 33342 and images were captured using an AMG EVOS Auto FL with a 40X objective and TRANS, GFP and DAPI lightcubes, Cells could also be bathed in PBS, fresh FCS-free media or other imaging buffers prior to viewing.

### Staining in Fixed Cells

Normal human fibroblast cells (TIG-1 cell line Coriell Cell Repository catalog number AG06173) were cultured in 96-well microplates (Greiner CELLSTAR^®^) using MEM/EBSS media containing 2 mM L-glutamine, Earle’s Balanced Salts (HyClone) and 5% FCS (Atlanta Biologicals) at 37°C in a 5% CO_2_ atmosphere to 60% confluency overnight. The media was removed and replaced with serum-free media containing 10 uM of the targeted lysosomal substrate 5(6)-(2-dimethylaminoethyl)carboxamido-2',7'-dichlorofluorescein diacetate (**5**) and the cells returned to the incubator as above for 1.5 hours after which time the media was removed by aspiration, and 100 uL of 50% MeOH, 4% formaldehyde and 1% glutaraldehyde in PBS added. The cells were fixed at 37°C in a 5% CO2 atmosphere for 15 minutes, the fixing solution aspirated and replaced with PBS containing 10ug/ml Hoechst 33342 and 1.9 uM TAMRA-SE, and the cells incubated for 1 hour. Finally the staining media was aspirated and replaced with PBS and images were captured using an AMG EVOS Auto FL with a 40X objective and TRANS, GFP and DAPI lightcubes.

### Measurement of Lysosomal Enzyme Activity in Cell Lysate

B-Lymphoblasts derived from a healthy donor (Coriell Cell Repository catalog number GM14643) and those derived from lysosomal storage disease patients (Coriell Cell Repository catalog numbers GM10870, GM10874 and GM06805) were harvested and centrifuged at 200xg for 5 minutes to pellet. Cells were resuspended in 200uL of reaction buffer (0.1M sodium citrate, 0.2M sodium phosphate, 0.1% triton X-100, 0.25% sodium taurocholate, pH 5.2). Lysate was cleared by centrifugation. Protein content was measured using Pierce BCA Assay kit (Thermo Fisher Scientific). Samples were then normalized to protein content and substrate (4-methylumbelliferyl β-D-glucopyranoside for Gaucher I cells or 4-methylumbelliferyl β-D-galactopyranoside for Krabbe cells (Marker Gene Technologies, Inc. Eugene, OR)) was added to a final concentration of 3mM. Samples were incubated at 37°C for 2 hours, and the reaction was terminated by the addition of 1mL of 400mM glycine (pH 10.8). Fluorescence was recorded using Tecan Infinite M200 Pro plate reader set to EX 360nm/ EM 449nm.

### Measurement of Lysosomal Enzyme Activity in Live Cells by Flow Cytometry

B-Lymphoblasts derived from a healthy donor (Coriell Cell Repository catalog number GM14643) and those derived from lysosomal storage disease patients (Coriell Cell Repository catalog numbers GM10870, GM10874 and GM06805) in serum free media were seeded at 50000 cells/well in 96 well round bottom plates (Corning, Corning NY). 5(6)-(2-dimethylaminoethyl)carboxamido-2',7'-dichlorofluorescein-3’,6’-di-O-β-D-glucopyranoside, octaacetate (**4**) was added to a final concentration of 5uM for Gaucher I cells or 5(6)-(2-dimethylaminoethyl)carboxamido)-2’,7’-dichlorofluorescein-3’,6’-di-O-β-D-galactopyranoside, octaacetate (**2**) was added to a final concentration of 10uM for Krabbe cells. Cells were incubated at 37°C/5% CO_2_ for 16 hours. DRAQ7^™^ (Immunochemistry Technologies LLC, Bloomington MN) was added to 3uM final concentration and the cells incubated at room temperature for 5 minutes. Cells were then run on a BD Accuri C6 Flow cytometer. Gates were set to exclude cellular debris and dead cells. Median FL-1 signal was measured on a gated population of viable cells.

### Measurement of Lysosomal Enzyme Activity in Live Cells by Microplate Assay

B-Lymphoblasts from healthy donors (Coriell Cell Repository catalog number GM07491) and those derived from Krabbe patients (Coriell Cell Repository catalog number GM06805) in serum free media were seeded at 200000 cells/well in 96-well black with optical bottom microplates (Nunc-Thermo Scientific 165305). 5(6)-(2-dimethylaminoethyl)carboxamido)-2’,7’-dichlorofluorescein-3’,6’-di-O-β-D-galactopyranoside, octaacetate (**2**) was added to a final concentration of 15uM. Cells were incubated at 37°C/5% CO_2_ for 16 hours. Fluorescence was then measured at ex 495nm/em 530nm using an EnVision Multilabel Plate Reader (Perkin Elmer, Waltham, MA). To account for potential differences in cell number, fluorescence was normalized to ATP content, measured by the addition of 25ul of CellTiter-Glo^®^ Reagent (Promega, Madison WI) at room temperature for 10 minutes and then reading of the luminescence on an EnVision Multilabel Plate Reader.

### Measurement of Lysosomal Enzyme Activity in Live Cells by Image Analysis

B-Lymphoblasts derived from healthy donors (Coriell Cell Repository catalog numbers GM14643 and GM07491) and those derived from lysosomal storage disease patients (Coriell Cell Repository catalog numbers GM10870, GM10874 and GM06805) were seeded at 50000 cells/well in 96 well glass bottom plates and allowed to settle overnight. Plates were centrifuged at 200xg for 5 minutes and media gently aspirated. Serum free media containing 5uM 5(6)-(2-dimethylaminoethyl)carboxamido-2',7'-dichlorofluorescein-3’,6’-di-O-β-D-glucopyranoside, octaacetate (**4**) for Gaucher I cells or 15uM 5(6)-(2-dimethylaminoethyl)carboxamido)-2’,7’-dichlorofluorescein-3’,6’-di-O-β-D-galactopyranoside, octaacetate (**2**) for Krabbe cells was added to the cells followed by incubation at 37°C/5% CO_2_ for 16 hours. Plates were centrifuged again and media aspirated. Cells were bathed in Opti-Klear^™^ Live Cell Imaging Buffer containing Hoechst 33342 and images captured on AMG EVOS Auto FL microscope fitted with 40X objective and GFP and DAPI lightcubes, Cells could also be bathed in PBS, fresh FCS-free media or other imaging buffers prior to viewing. Captured images were then quantified using Cell Profiler (Broad Institute, Boston MA). Objects in the blue and green channels were identified using Global Otsu Two-Class thresholding method and the intensity of the pixels contained within those objects measured for the green channel and the number of objects measured for the blue channel. The average green intensity per cell was calculated for each image and the average per cell over all images calculated.

## Results

### Colocalization Studies

**Fig 2:**
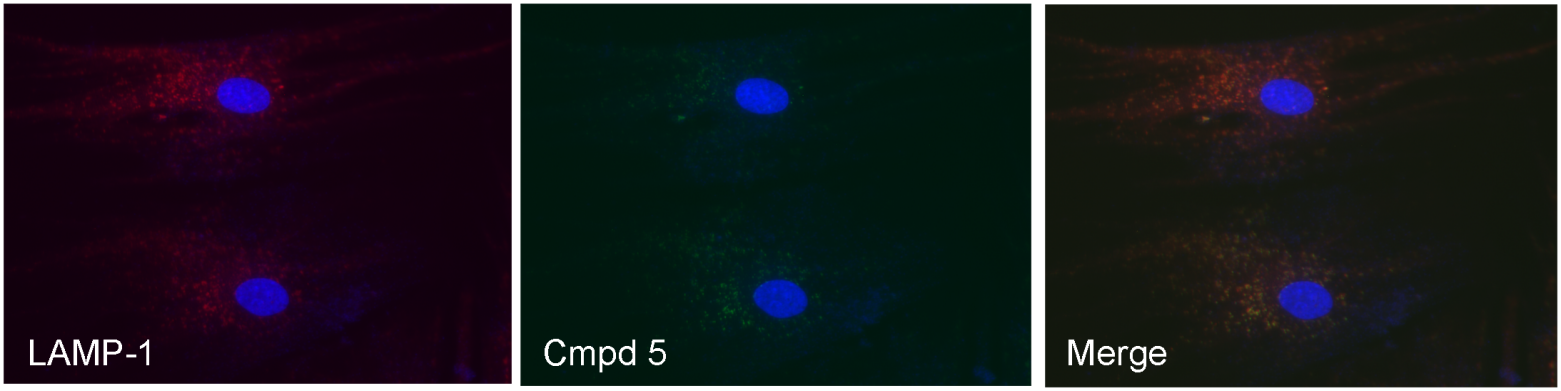
Localization of Targeted Lysosomal Substrate and anti-LAMP1 Antibody. Colocalization of the targeted lysosomal substrate **(5)** with anti-LAMP1 antibody staining was measured in AG06173 live human fibroblasts by incubation with 5uM 5(6)-(2-dimethylaminoethyl)carboxamido-2',7'-dichlorofluorescein diacetate (**5**) for 1 hour, followed by fixing the cells, permeabilization and staining with a primary rabbit anti-LAMP1 pAb antibody at 1.6ug/mL and incubation at room temperature for 1 hour. After washing, a secondary Alexa Fluor^®^ 555 conjugated Goat Anti-Rabbit antibody was added and incubated for 1 hour at room temperature. Cells were washed 3 times in PBS containing Hoechst 33342 added to the second wash and imaged. Fluorescence staining of nuclei (blue) and lysosomes with both anti-LAMP1 (red) and **(5)** (green) was captured using fluorescence microscopy. Merging of green and red images demonstrated >99% degree of colocalization between the two stains.

**Fig 3:**
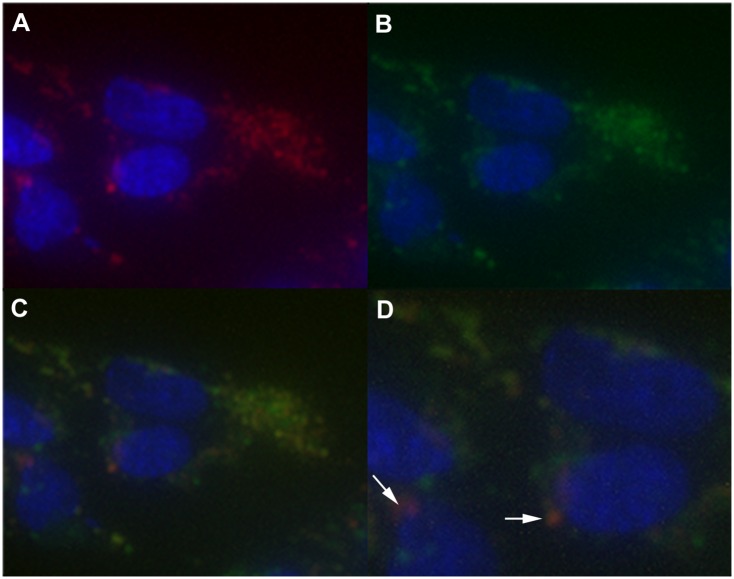
Localization of Targeted Lysosomal Substrate and LysoTracker^®^ Red. Colocalization (C) of the targeted lysosomal substrate **(4)** (B) and LysoTracker^®^ Red (A) was measured by incubation with 5uM **(4)** for 16 hours followed by incubation with 75nM LysoTracker^®^ Red DND for 30minutes. Cells were washed with PBS and bathed in Opti-Klear^™^ Live Cell Imaging buffer containing 10ug/mL Hoechst 33342 prior to imaging using an AMG EVOS Auto FL with appropriate lightcubes. Fluorescence analysis of LysoTracker^®^ Red (red) and **(4)** (green) exhibited a high degree of co-localization between the two stains in nearly all cases, with LysoTracker^®^ occasionally staining some additional structures indicated by arrows in panel D.

The targeted esterase substrates (**5**) and (**6**) were examined for their ability to specifically partition to intact lysosomes in living cells by comparing their staining patterns to that of known lysosome probes including LAMP1 antibody staining and LysoTracker^®^ Red DND-99 staining. As seen (Figs 2 and 3) both the LAMP1 antibody staining patterns and the LysoTracker^®^ Red DND-99 staining distribution were found to co-localize with the esterase substrates (**5**) and (**6**), exhibiting a high degree of co-localization in all cases. LysoTracker^®^ Red DND-99 exhibited some additional staining in other acidic vesicles, most likely early phagocytic, autophagosome and endosome vesicles, as is documented in the literature [6,7]. The esterase substrates presumably exhibit somewhat more specific staining patterns as their distribution is linked to enzymatic activity present in lysosomal or mature fused lysosomal organelles rather than merely due to pH partitioning. Similar, albeit less defined colocalization was observed with the other lysosomal stains, such as acridine orange and neutral red (data not shown).

### Enzyme Inhibition

To confirm that the substrates are monitoring enzyme activity, a series of inhibition experiments were performed. First, chloroquine, a lysosomotropic agent typically found as its diphosphate salt (pKa 8.5) was examined as a general inhibitor of lysosomal enzyme activity. The unprotonated form of chloroquine rapidly diffuses across cell and organelle membranes where it preferentially accumulates in lysosomes. Once in the lower pH environment of the lysosome (pH 4.6) chloroquine becomes protonated and unable to diffuse again across the lysosomal membrane [8], acting to raise the intralysosomal pH [9]. This accumulation has also been shown to lead to general inhibition of lysosomal enzymes [10], as well as preventing fusion of endosomes and lysosomes. Interestingly, treatment with chloroquine has also been shown to result in the increased number and size of lysosomes as well as autophagosomes as monitored by LysoTracker^®^ staining [6] most likely due to buildup of unprocessed substrates in the lysosomes.

**Fig 4:**
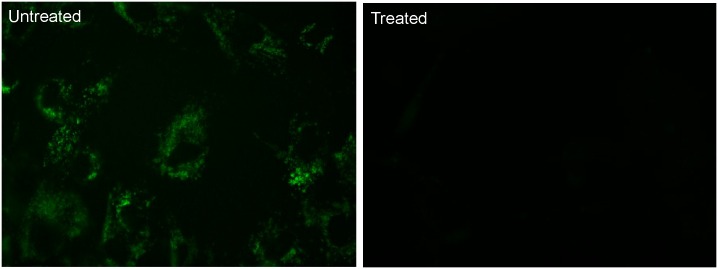
Inhibition of Lysosomal Metabolism by Chloroquine Leads to Reduction in Lysosomal Staining. Healthy donor fibroblasts were incubated for 16 hours with or without 300uM chloroquine at 37°C/5% CO_2_. The media was removed and replaced with serum free media containing 10uM 5(6)-dimethylaminoethylcarboxamido-2',7'-dichlorofluorescein diacetate (5) and cells were incubated for 2 hours. Staining media was removed and cells were washed 3 times with PBS prior to the addition of Opti-Klear^™^ Live Cell Imaging Buffer. Images were then captured on a Zeiss Axio Observer A1 inverted microscope fitted with a 40X lens and FITC filter set. After treatment with chloroquine, staining in lysosomes is significantly reduced (right panel).

Since the targeted lysosomal substrates only become fluorescent upon enzymatic activity, treatment of cells with chloroquine should act to inhibit enzymatic activity and reduce staining. Fig 4 illustrates staining patterns after treatment with chloroquine where combined application of the esterase substrate (**5**) exhibited significantly reduced staining in lysosomes despite increased lysosomal size. This data indicates the esterase substrate is not simply staining lysosomes, as with LysoTracker^®^ dyes, but is useful in monitoring changes in intracellular enzyme activity upon inhibition.

**Fig 5:**
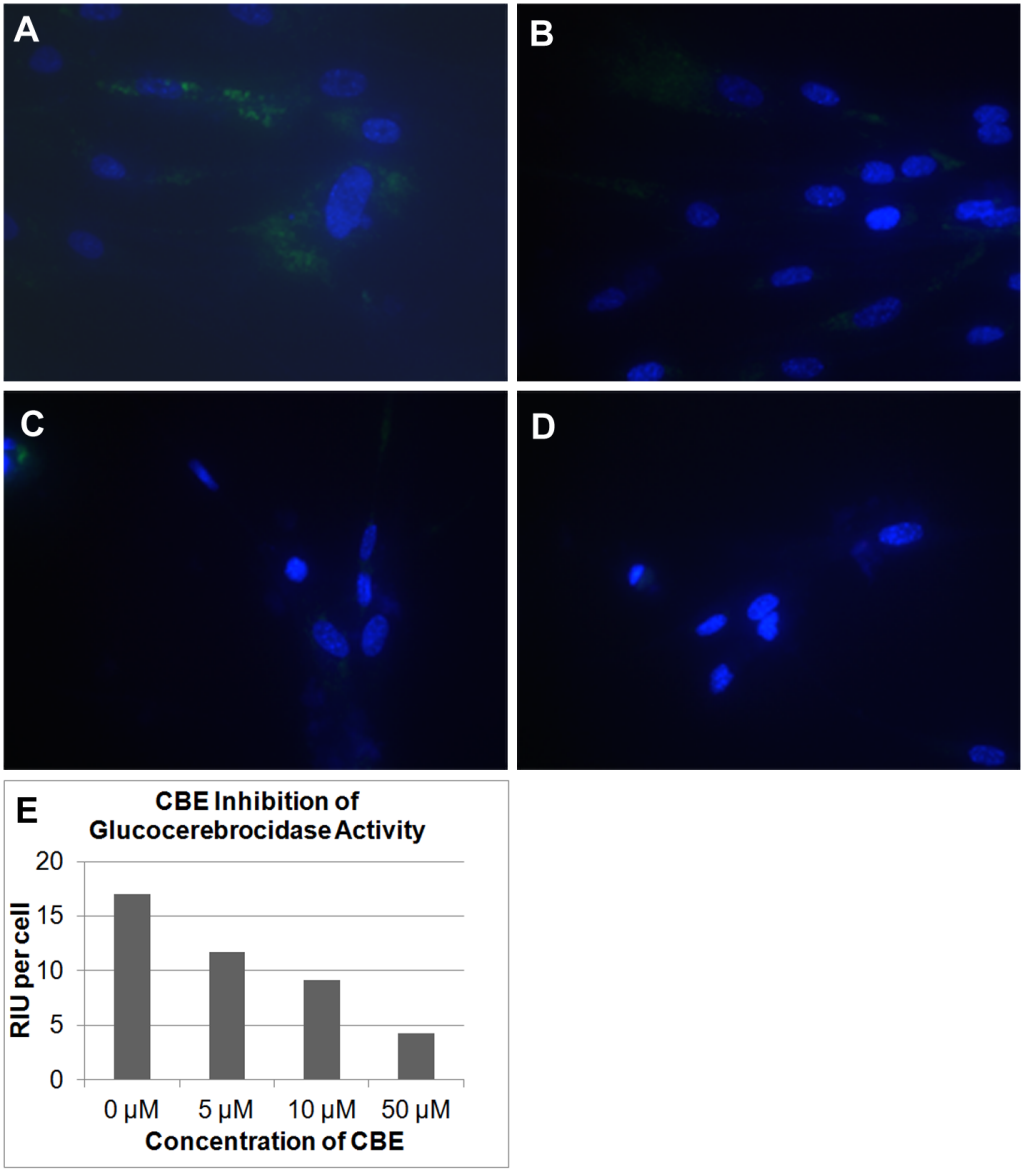
Inhibition of Lysosomal Glucocerebrosidase by CBE Leads to Reduction in Lysosomal Staining. Healthy donor fibroblasts AG06173 were incubated for 16 hours with 0(A), 5(B), 10(C) and 50uM (D) of Conduritol β-Epoxide (CBE), then treated with 5uM 5(6)-(2-dimethylaminoethyl)carboxamido-2',7'-dichlorofluorescein-3’,6’-di-O-β-D-glucopyranoside, octaacetate (4) at 37°C/5% CO_2_ for 16 hours. The media containing the β-glucosidase substrate and inhibitor was removed and the cells washed 3 times with PBS prior to the addition of Opti-Klear^™^ Live Cell Imaging Buffer containing 10ug/ml Hoechst 33342. Images were captured using an AMG EVOS Auto FL with a 40X objective and GFP and DAPI lightcubes. Intensity of green fluorescent signal was quantified using Cell Profiler (E). As the inhibitor concentration increases, a relativel reduction in staining is observed.

In a similar manner conduritol-beta-epoxide (CBE) was employed as a selective, irreversible β-glucosidase inhibitor. CBE has been widely used to inhibit the lysosomal glucosylceramide-degrading enzyme (glucocerebrosidase, GBA) in live cells and has even been used as a method of mimicking certain genetic metabolic disorders such as Gaucher disease [11, 12]. As seen in Fig 5, treatment of healthy donor fibroblast cells with CBE at different concentrations exhibited a dose-response change in staining using the lysosomal targeted β-glucosidase substrate (**4**) indicating that this targeted glucosidase substrate is capable of monitoring lysosomal β-glucosidase enzyme activity and measuring residual levels of enzyme activity after inhibition. Residual activity at higher concentrations may be attributed to background detection during cell profiler analysis as no staining is visible in the images prior to analysis, it has also been reported that after similar 16 hour incubation periods there is still some residual GCase activity [13].

### Membrane Permeabilization

Traditional fluorogenic substrates used to monitor lysosomal enzyme function, such as 4-methylumbelliferyl β-D-glucopyranoside (MUGlc) for acid β-glucosidase, 4-methylumbelliferyl β-D-galactopyranoside (MUGal) for β-galactosidase or similar MUG type substrates for other glycosidase enzymes [14] typically employ lysis assay methods or other cell disruption protocols, such that their use in live cell analysis is limited. Attempts to stain live fibroblast or leukocyte cell lines with the targeted lysosomal glycosidase substrates for β-galactosidase and β-glucosidase, compounds (**1**) and (**3**) respectively, produced minimal or diffuse staining.

**Fig 6:**
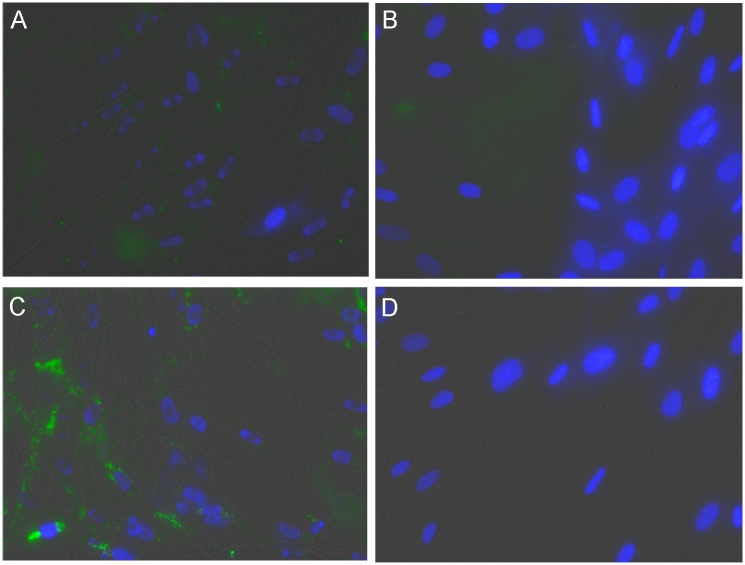
Permeabilizing Groups are Required for Efficient Entry of Substrates into Cells. Healthy donor fibroblasts were incubated for 16 hours with 5(6)-(2-dimethylaminoethyl)carboxamido)-2’7’-dichlorofluorescein-3’,6’-di-O-β-D-glucopyranoside (3) (B,D) or 5(6)-(2-dimethylaminoethyl)carboxamido-2',7'-dichlorofluorescein-3’,6’-di-O-β-D-glucopyranoside octaacetate(4) (A,C), at 12.5uM (A,B) and 25uM (C,D). Prior to imaging staining media was removed and cells washed 3 times with PBS. Cells were then bathed in Opti-Klear^™^ Live Cell Imaging Buffer containing 10ug/ml Hoechst 33342 and images were captured using an AMG EVOS Auto FL with a 40X objective and TRANS, GFP and DAPI lightcubes. Only the targeted substrate with the peracetate permeabilizing groups exhibited significant staining.

It is recognized, however, that peracetylated sugars can more easily pass through the outer and inner cell membranes of live cells (Laughlin, et al., 2007) where they can be utilized by intracellular biosynthetic machinery. Staining with the peracetylated versions of the substrates, compounds **(2)** and **(4)**, provided specific, punctate staining patterns in live cell format, as is shown in Fig 6. Ubiquitous intracellular esterase activity in the lysosome is presumably able to remove the acetate permeabilizing groups releasing the free sugar which is then available for measurement of desired glycosidase activity in the cell. Although the enzyme reaction kinetics are somewhat slower using these peracetate versions of the substrates, careful monitoring of time versus staining intensities can be used to provide effective quantitative data of relative enzyme activity.

### Cell Imaging after Fixation

**Fig 7:**
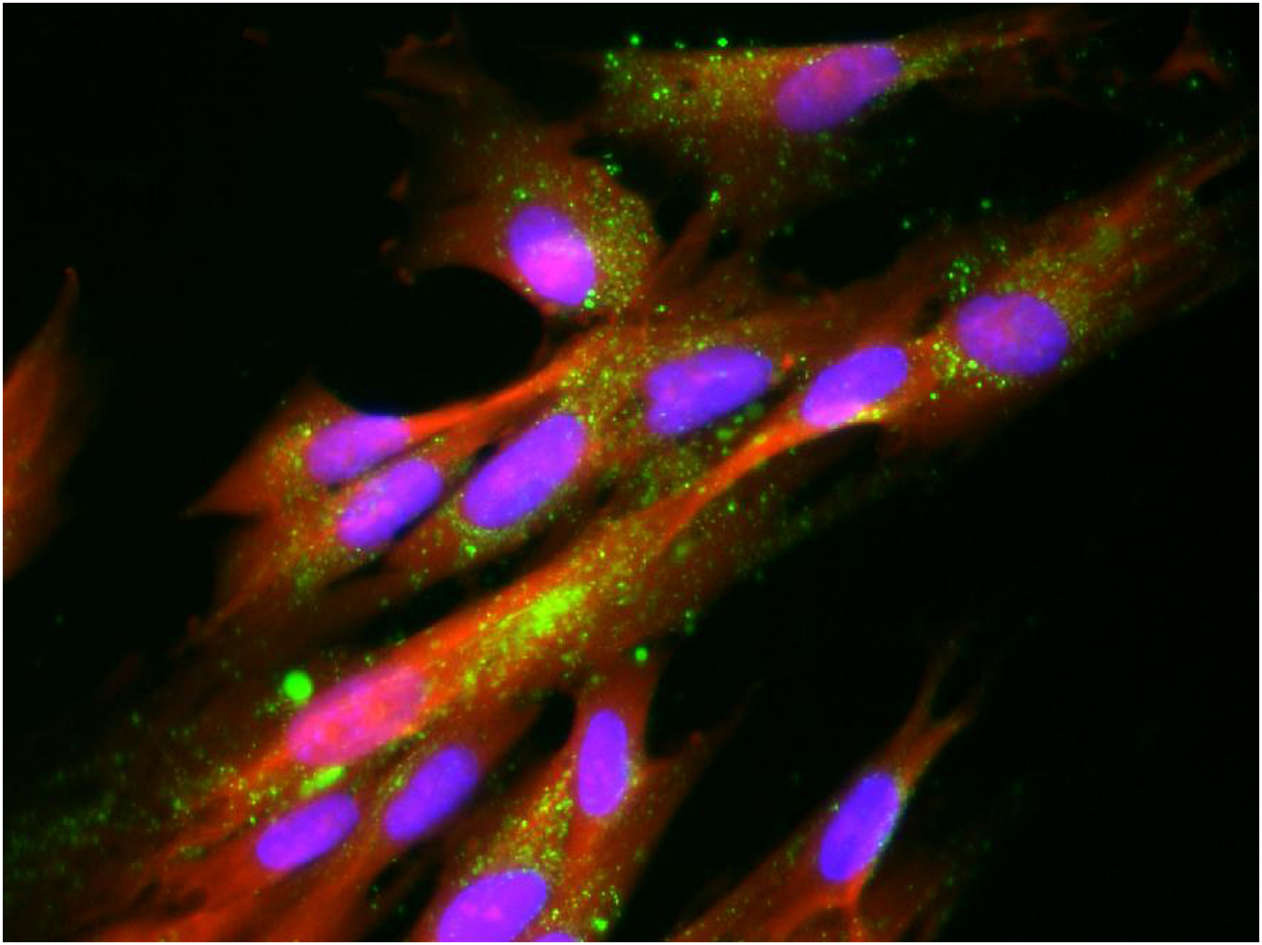
Triply Labeled Fixed AG06173 Fibroblasts. Normal human AG06173 fibroblast cells were incubated with 10 uM of the lysosome targeted esterase substrate 5(6)-(2-dimethylaminoethyl)carboxamido-2',7'-dichlorofluorescein diacetate (**5**) for 1.5 hours after which time the media was removed and the cells were fixed. The fixing solution was aspirated and replaced with PBS containing 10ug/ml Hoechst 33342 and 1.9 uM TAMRA-SE for 1 hour followed by imaging using an AMG EVOS Auto FL with a 40X objective and TRANS, GFP and DAPI lightcubes. Lysosome (green), nucleus (blue) and cell membrane (red) staining were examined.

Cell fixation and permeabilization techniques have been developed to help allow analysis of internal structures and biological molecules using techniques such as immunohistochemical staining with antibodies, fluorescent labeling of nucleic acid and RNA, labeling internal structures for clinical diagnosis of infectious diseases, whole cell ELISA or Western analyses and allied techniques. Fixation can also trap the cells at a specific state of differentiation or growth and is therefore particularly useful in high-throughput screening applications. Fluorescent probes that can withstand standard fixing and permeabilization conditions are therefore of added utility. Since the LysoTracker^®^ probes have been shown to be amenable to fixation conditions [15], the ability of the targeted lysosomal substrates to withstand fixation (4% formaldehyde/1% glutaraldehyde in PBS) and permeabilization (0.2% Triton-X-100 in PBS) conditions was examined. Fig 7 shows a triple labeling experiment wherein the cell lysosomes were labeled with (**5**) for 1.5 hours and then fixed and co-stained with Hoechst 33342 (blue) to label the nuclei as well as TAMRA-SE (red) to label cell membranes. Staining of the lysosomes remained robust even after the fixation and permeabilization conditions of this method. Similar fixing and permeabilization prior to staining with primary and secondary antibodies (see Fig 2) were also found to be amenable to the substrate staining procedure. The preferred method was to label for the lysosomal enzymes prior to fixing, since the fixation conditions may affect enzyme activities.

### Measurement of Lysosomal Enzyme Activity in Disease Cell Lines

**Fig 8:**
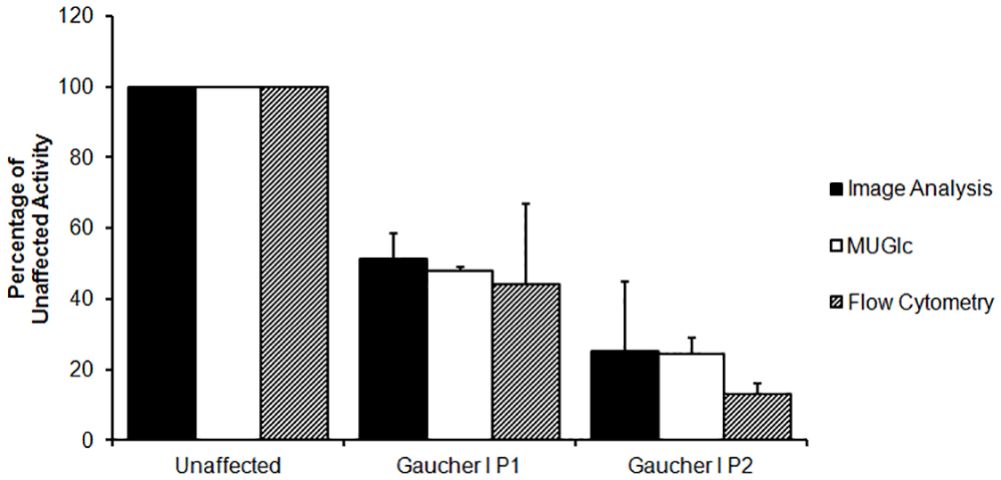
Lysosomal beta-Glucocerebrosidase Activity Measured by Multiple Methods. Glucocerebrosidase activity in healthy (GM14643) and two Gaucher I cell lines (GM10870,GM10874 containing the N370S mutation) was measured by staining with (**4**) and image analysis by flow cytometry as well as by using a standard 4-methylumbelliferyl β-D-glucopyranoside (MUG) lysis assay. Enzyme activity measurements are shown to be consistent when measured using (**4**) in two different formats when compared to the standard diagnostic assay using MUG.

Gaucher I Disease is a lysosomal storage disease characterized by mutations in the GBA1 gene expressing beta-glucocerebrosidase causing a deficiency in this enzyme in the lysosome. Measurement of lysosomal β-glucosidase enzyme activity in Gaucher I cells is diagnostic of the disease and is usually performed by cell lysis techniques using analysis with MUG (4-methylumbelliferyl β-D-glucopyranoside). The MUG assay is performed at low pH with added sodium taurocholate to provide specificity for the lysosomal glucosidase (GCase) levels. Staining data obtained by incubating Gaucher I cells with the targeted β-glucosidase substrate (**4**) followed by image analysis using the CellProfiler program [16] with data obtained by standard MUG assay [17] as well as with data obtained from staining with **(4)** and analysis by flow cytometry (Fig 8), demonstrated that the levels of staining using the new lysosomal targeted substrate were in good correlation with those obtained using the other two methods, supporting the hypothesis that the targeted substrates measure enzyme levels in the lysosome. All data was normalized to that obtained using the same staining methods in cells obtained from a normal patient.

**Fig 9:**
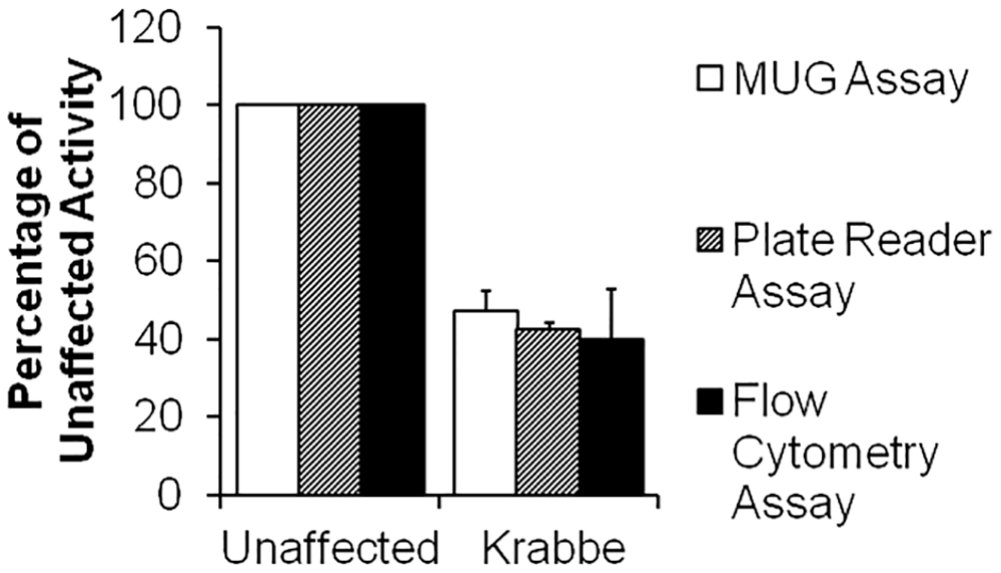
Lysosomal Galactosylceramide Activity Measured by Multiple Methods. Galactosylceramide activity in Normal and Krabbe cells was measured using the standard MUG assay and using staining with (**2**) in both imaging and flow cytometry formats.

**Fig 10:**
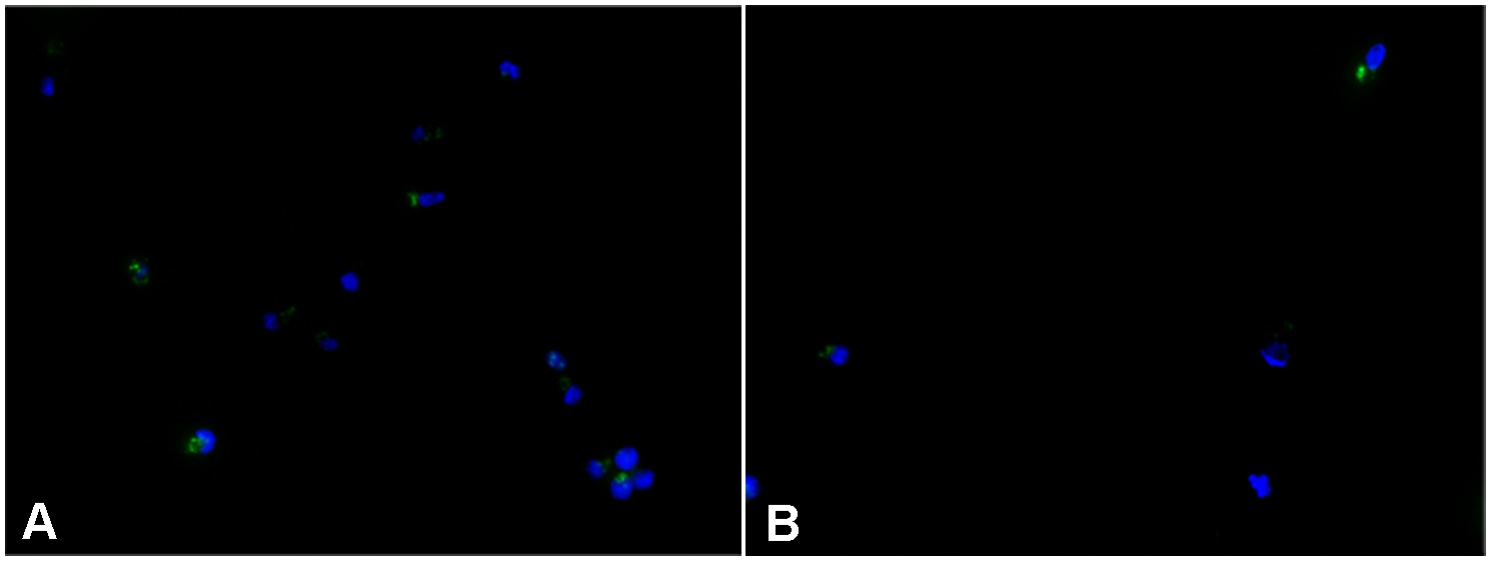
Staining for Lysosomal Galactosylceramidase Activity in B-Lymphocytes. B-Lymphoblasts from a healthy donor and those derived from a Krabbe patient were seeded and media containing 15uM 5(6)-(2-dimethylaminoethyl)carboxamido)-2’,7’-dichlorofluorescein-3’,6’-di-O-β-D-galactopyranoside, octaacetate (**2**) added with incubation for 16 hours. Cells were co-stained in Opti-Klear^™^ Live Cell Imaging Buffer containing Hoechst 33342 and images captured on AMG EVOS Auto FL microscope fitted with 40X objective and GFP and DAPI lightcubes. Targeted lysosomal substrate exhibited reduced staining for Krabbe patient cells when compared to staining in cells from healthy individuals.

In a similar manner, measurement of lysosomal β-galactosidase activity was performed using B-Lymphoblasts from a healthy donor and those derived from lysosomal storage disease patients with Krabbe Disease. Analysis by staining with the lysosome targeted β-galactosidase substrate (**2**), using standard Krabbe 4-MUGal analysis [18] after cell lysis, by fluorescence microplate assay and by using flow cytometric analysis are shown in Fig 9. Again, the enzyme activity levels of the three methods showed good correlation. Representative staining images with (**4**) of normal healthy and Krabbe disease patient leukocytes are shown in Fig 10.

### Measurement of Lysosomal Enzyme Activity with Alternative Fluorescent Substrates

**Fig 11:**
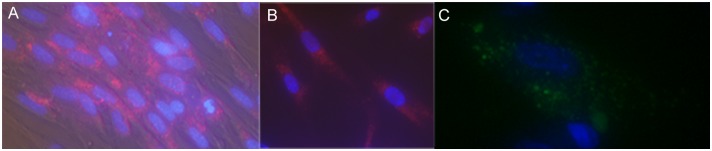
Analysis of Lysosomal Enzyme Activity Using Alternative Fluorescent Substrates. AG06173 human fibroblast cells were stained with 10 uM of an esterase substrate with an alternative morpholino based targeting group (6) (C), 10uM of the resorufin based β-glucosidase substrate (9) (B), or 10uM of the resorufin based esterase substrate (10) (A) followed by incubation at 37°C in 5% CO_2_ atmosphere for 16 hours. The cells were then washed with pre-warmed PBS followed by addition of Opti-Klear^™^ Live Cell Imaging Buffer containing 10ug/ul Hoechst 33342). Cells were imaged using an AMG EVOS Auto FL Fluorescence Microscope equipped with appropriate lightcubes and 40X air objective.

In order to expand the utility of these substrates an alternative targeting group was examined **(6)** for lysosomal localization (Fig 11). Additionally, in order to provide for multiplexed assays allowing for study of multiple enzymes within the same cell, substrates **(9)** and **(10)** derived from the red emitting fluorophore resorufin were developed. Both show the expected localization and activity (Fig 11).

## Discussion

We have developed a series of targeted lysosomal enzyme substrates based upon fluorophores with low pKa values for optimum fluorescence in the low pH environment found in the lysosome. In addition, these fluorogenic substrates contain a weakly basic targeting group, similar to that used with other lysosomal stains such as the LysoTracker^®^ dyes, that acts to sequester their localization to this organelle. Finally, these substrates contain an enzyme cleavable group that is specific for the enzyme being examined. Further permeabilizing groups on these substrates were found to help with cell loading as well as localization to the target organelle.

The addition of the enzyme cleavable group renders these fluorogenic substrates either non-fluorescent, in the case of fluorescein-based substrates, or weakly fluorescent (with shifted emission) in the case of the resorufin-based substrates. This property makes them ideal for quantitative measurement of activity and potentially even kinetic analysis of defective or mutant enzyme reaction rates inside living cells. Since these new substrates can be produced from multiple fluorophores, each with well separated excitation and emission spectral properties, the promise of multiplexed analysis of several enzyme activities simultaneously within the same cells becomes feasible. Because individual genetic mutations do not always directly correspond to disease progression, these methods hold the promise of measuring lysosomal enzyme activity to study the effect of a mutation or the relationship between enzyme activity and disease progression between several patient samples, in real time, and potentially relating this data to more precise treatment regimens.

We were surprised to find that, for measurement of glycosidase activities, use of the native sugars for conjugation in the substrates (e.g. (**1**) and (**3**)) was found to result in limited staining, even after overnight incubation. Additional permeabilization techniques were required. It is known in the literature that acetate-protected sugar moieties can more easily pass across the cell membrane [19], where they accumulate inside the cell and are deblocked by the action of ubiquitous intracellular esterases. Using this method, we were able to increase staining for the glycosidase substrates by use of peracetate protected glycosidase substrates. In addition, peracetate substrates were found to be more stable in cell culture conditions and even seemed to show greater organelle specificity. Excellent staining with (**5**) and (**6**) indicated that there was abundant esterase activity in the lysosomes, even in disease cell lines.

An interesting question surfaced concerning trafficking of the substrates in the cell and the order of activity. Since there are known to be additional glycosidase or esterase activities outside of the lysosome, it is of interest to evaluate whether the targeted substrates are partitioned to the lysosome prior to enzymatic release of the fluorophores, or whether enzyme activities in the cytosol or other organelles can produce the targeted dyes which are then partitioned to the lysosome.

Three lines of support seem to point to the former mechanism, namely that the intact substrates are quickly partitioned first to the target organelle where the enzyme activity of interest releases their fluorescent signal. First, our attempts to stain cells, even at very high concentrations, with the base targeted dyes (**7**) and (**11**) resulted in essentially no staining. Apparently, due to the positive charge of the targeting group, these base dyes are not very permeant to the cell membrane. Secondly, time lapse photomicroscopy (S1 Fig) of the staining patterns observed for these substrates exhibited steadily increasing fluorescence staining specifically in the lysosomes, with virtually no cytosolic or other organelle staining. Finally, the correlation of the staining levels to those obtained by independent techniques measuring lysosomal enzyme activities, indicate that the substrates appear to be predominantly measuring the enzyme activity in the target organelle and not cell-wide activities.

A microplate based assay system was developed to utilize intact, living cell lines and provide an accurate analysis of enzyme activity for high content screening. Originally our intention was to use adherent cells for analysis, since they would be amenable to washing and staining protocols. However in order to obtain an appropriate number of cells for analysis in a timely and effective manner we chose to instead use immortalized B-Lymphoblast cells [20] for our primary analysis and restrict use to fibroblasts to those analyses requiring detailed imaging.

Because the leukocytes are much smaller than fibroblasts, it was necessary to place many more cells in each well in order to get acceptable signal intensities over noise; and as they are non-adherent there was a need for a no-wash, simple additive assay. Washing steps requiring removal of supernatant media often led to loss of cells. Also, since leukocytes are known to clump in culture, we utilized several declumping techniques including physical re-pipetting of culture solutions to remove more dense clumps as well as addition of Accutase. In order to count the cells *in situ* as well as determine cytotoxicity, a CellTiter-Glo^®^ assay was employed. Finally, we observed that some few leukocytes in each well routinely exhibited very high staining levels, which we attributed to possible ongoing cell division where those cells exhibited increased number, size and enzyme activity in their lysosomes. We found that they were more amenable to growth in large numbers in smaller volume flasks, presumably due to release of certain growth co-factors at higher density. CellProfiler analysis, however, was made more difficult because of the small cell size and clumping properties of the leukocytes.

The use of leukocytes, however, was advantageous in flow cytometry, since the cells are small and do not need preparation (trypsinization, etc.) prior to analysis. In addition, the cytometric data obtained was routinely considered to be more accurate than microplate results, since dead or dying cells were channel separated by co-staining with DRAQ7^™^, a membrane impermeant far-red nucleus staining fluorescent dye. And since the data is averaged based on staining of individual cells, plate reader artifacts can be reduced. The effects of other cell states, such as apoptotic, necrotic, cells undergoing replication or antibody staining for specific cell types could also be sorted or co-analyzed. Flow cytometric analysis may potentially offer the most accurate prospect for use of these substrates for diagnostic analysis of disease progression or even response to treatment methodologies.

**Fig 12:**
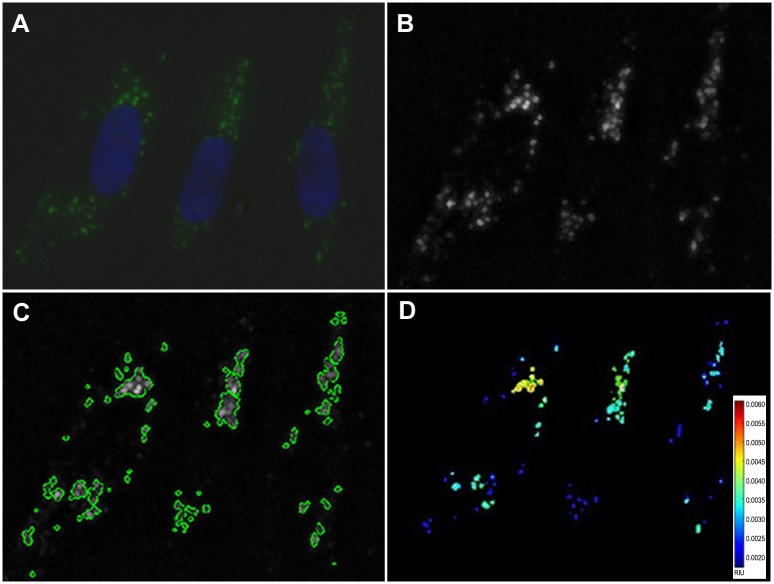
Image Analysis of Stained Cells using Cell Profiler. AG06173 human fibroblast cells were stained with the targeted lysosomal substrate **(4)** (green) and Hoechst 33342 nuclear stain (blue). Captured images (A) were then quantified using Cell Profiler image analysis. Objects in the blue and green channel images were identified using Global Otsu Two-Class thresholding method (B,C) and the intensity of the pixels contained within those objects measured for the green channel and the number of objects measured for the blue channel. The average green intensity per cell was calculated for each image and the average over all images. The intensity of the selected objects was measured in relative intensity units (RIU) and is represented as a heat map (D).

CellProfiler image analysis (Broad Institute) of the cells after staining was routinely employed as a method of final assessment of enzyme activity (Fig 12). However, setting up the pipelines for this method involved determining certain parameters that could change in each experiment. In order to validate the data, three image sets were first analyzed manually to ensure that primary object identification, or segmentation, of the nuclei was occurring accurately. The pipeline had to be adjusted for the variation of staining intensity or microscope light intensities used in each experiment, and was addressed by adding illumination correction modules in the pipeline. The images rendered from the illumination correction were then used to allow the segmentation of objects of interest. Data was interpreted as a percentage of the healthy cells’ staining intensity on a per cell basis, using Hoechst nuclear staining to quantify cell number (nuclei) and the bright-field image to select the outer edge of each cell. The cell edges were identified by first transforming the bright-field image into a binary image, which separated the foreground from the background. The nuclei were identified using the global maximum correlation thresholding (MCT) method and clumped objects were distinguished with a Laplacian of Gaussian (LoG) transformation.

The same illumination correction and threshold parameters placed upon the nuclear images were applied to the images containing the staining of interest to segment the punctate staining and isolate it from the background. After identifying the staining regions of interest, the objects’ intensities were measured within the confines of the identified regions, but using the original images. Fibroblasts and leukocytes required slightly different pipelines because of the differences in the size of their nuclei and general cell shape. Once a pipeline was developed, it was used for analysis of all cells in that experiment in order to ensure accurate correlation.

## Conclusion

Intracellular enzyme activities are known to be involved in numerous metabolic activities as well as being implicated in various disease states. Defects in lysosomal enzymes have been associated with a number of genetic diseases. These include mutations within the GBA1 gene encoding acid beta-glucosidase involved in Gaucher Disease as well as deficiency of lysosomal β-galactosidase in Krabbe disease. Recent evidence has also implicated a link between the levels of glucocerebrosidase activity (GCase) and disease progression in other diseases, such as Parkinson’s Disease. But methods to determine intracellular enzyme activities using a live cell format for diagnosis or for drug discovery are limited by often involved and complex cell preparation methods or are based upon selective inhibition, indirect or special analysis techniques. These often preclude their use in high-content or high-throughput analysis methods. We have developed a series of targeted enzyme substrates that can be used in a live cell format to measure lysosomal enzyme activities. We believe the use of the new lysosomal targeted substrates in a live-cell, HCS/HTS platform could be a valuable tool in the search for new therapeutics, not only for Gaucher I and Krabbe diseases, but also other metabolic diseases involving lysosomal enzymes.

## Supporting Information

S1 Fig Time lapse of Lysosomal Staining. Long-term video microscopy of the staining of AG06173 cells using compound **(4)** (5 uM final concentration) overnight, captured using an AMG EVOS Auto FL microscope fitted with 40X objective and GFP lightcube.
(MP4)

S1 Text Chemical Synthesis of Compounds. (DOCX)

## Abbreviations

EX: excitation
EM: emission
CH_2_Cl_2_: dichloromethane
MeOH: methanol
Rf: retention factor
TLC: thin-layer chromatography
aq: aqueous
HCl: hydrochloric acid
CH_2_Cl_2_: dichloromethane
CDCl_3_: deuterochloroform
Ac: acetate
HOAc: acetic acid
SiO_2_: silica
EtOAc: ethyl acetate
DMF: dimethylformamide
NMR: nuclear magnetic resonance
DMSO: dimethylsulfoxide
HPLC: high-pressure liquid chromatography
