# Supplementary material for: Fluorogenic Substrates for Visualizing Acidic Organelle Enzyme Activities
Source: PLoS One. 2016 May 26;11(5):e0156312. doi: 10.1371/journal.pone.0156312 (PMC4882035; doi:10.1371/journal.pone.0156312)
Supplement: S1 Text — (DOCX) [file pone.0156312.s002.docx]

S1 Text

Materials

Chemical reagents were obtained from Sigma-Aldrich-Fluka Chemical Co. (St. Louis, MO) unless otherwise described and were used without further purification. HCl, acetic anhydride, and dry pyridine were obtained from Mallinckrodt Chemicals (Phillipsburg, PA). Hydrobromic acid (33 wt % solution in glacial acetic acid), silver carbonate, Amberlite IRC-50 ion-exchange resin, sodium methoxide (25 wt % solution in MeOH), and anhydrous CH_2_Cl_2_ were obtained from Aldrich Chemical Co. (Milwaukee, WI). Sym-collidine was from Acros Organics (Morris Plains, NJ). All chemicals were used without further purification. Opti-Klear^TM^ Imaging Buffer is a HEPES based neutral-osmolarity imaging buffer (Marker Gene Technologies, Inc., Eugene, OR). ^1^H-NMR spectra were obtained using a Varian Inova 300-MHz nuclear magnetic resonance instrument.

Synthesis of 5(6)-(2-dimethylaminoethyl)carboxamido)-2’7’-dichlorofluorescein (7)

To a dry flask under anhydrous N_2_(g) conditions was added *N*-hydroxysuccinimide (5.75 g, 50 mmol) and trifluoroacetic anhydride (20.0 mL, 144 mmol) and this mixture was allowed to stir at room temperature for 1.5 h. The reaction mixture was evaporated under reduced pressure, co-evaporating with dry toluene (3 x 20 mL) at 50^o^C and dried *in vacuo* overnight to give O-trifluoroacetyl-N-hydroxysuccinimide as a white amorphous crystalline solid (10.57g, 100%).

Under anhydrous conditions, a sample of O-trifluoroacetyl-N-hydroxysuccinimide (10.57 g, 50 mmol) was dissolved in anhydrous DMF (20 mL) and 5(6)-carboxy-2’,7’-dichlorofluorescein (5.20 g, 12 mmol, prepared by reaction of 4-chlororesorcinol with trimellitic anhydride in methanesulfonic acid) and dry pyridine (10.0 mL, 130 mmol) were added. This mixture was allowed to stir at room temperature under anhydrous conditions overnight. The reaction mixture was then poured into ice-water (300 mL) with stirring and extracted with EtOAc (200 mL). The aqueous layer was extracted again with fresh EtOAc (100 mL) and the combined organic layers were washed with H_2_O (200 mL) and brine (200 mL), dried over anhydrous sodium sulfate, filtered, evaporated and dried *in vacuo* to give 5(6)-carboxy-2’,7’-dichlorofluorescein, NHS ester as a bright orange amorphous solid (4.65 g, 84%). Chromatography (TLC: SiO_2_ plate, 7:3 EtOAc:MeOH irrigant, Rf = 0.53 and 0.79) indicated approximately a 1:1 mixture of the two isomeric active esters.

A sample of the above NHS-esters (4.65 g, 10.1 mmol) was dissolved in anhydrous DMF (50 mL) and *unsym*-*N,N*-dimethylethylenediamine (1.65 mL, 15 mmol) added. This mixture was allowed to stir under anhydrous conditions, at room temperature overnight. EtOAc (200 mL) was added with stirring for 30 min. and the pale orange precipitate filtered, washed with EtOAc and dried *in vacuo* to give the title mixture of 5(6)-dimethylaminoethyl amides (2.88 g, 57%). The combined EtOAc filtrates from above were extracted with 1N HCl/H_2_O solution (2 x 100 mL) and the resulting aqueous layers were washed with CH_2_Cl_2_ (2 x 25 mL), and evaporated to dryness. The resulting orange oil was triturated with diethyl ether to give a second crop of the title compound (as the HCl-salt form) (2.20 g, combined yield = 97%). TLC (irrigant = 3:3:3:1 CH_2_Cl_2_:MeOH:H_2_O:HOAc, Rf = 0.11 and 0.19(5- and 6-isomers); ^1^H-NMR (300 MHz, DMSO-*d*_6_) δ:8.77(t, 0.5H); 8.63(t, 0.5H); 8.15 (m, 2H); 7.73 (br s, 0.5 H); 7.40 (d, 0.5 H); 6.72 (s, 4H); 6.53(br s, 1H); 3.58 (q, 1H); 3.42 (q, 1H); 2.84 (t, 1H); 2.68 (t, 1H); 2.35 (s, 6H).

Synthesis of 5(6)-(2-dimethylaminoethyl)carboxamido)-2’,7’-dichlorofluorescein-3’,6’-di-O-β-D-galactopyranoside, octaacetate (2).

A sample of 5(6)-(2-dimethylaminoethyl)carboxamido)-2’7’-dichlorofluorescein (3.20 g, 6.37 mmol) was suspended in a mixture of anhydrous CH_2_Cl_2_ (40 mL), anhydrous THF (40 mL) and anhydrous acetonitrile (20 mL). To this solution was added acetobromogalactose (6.76 g, 16.4 mmol), dry silver carbonate (2.20 g, 7.96 mmol) and sym-collidine (2.0 mL, 15.1 mmol), the flask covered in Al-foil (darkness) and allowed to stir at ambient temperature under anhydrous N_2_(g) for 72 h. Additional acetobromogalactose (5.5 g) and silver carbonate (1.75 g) were added and the reaction continued stirring as above for 18 h. The reaction mixture was filtered through a Celite^TM^ pad and the filtered silver salts washed with excess CH_2_Cl_2_. The filtrates were combined and evaporated to a brown oil, redissolved in CH_2_Cl_2_ (100 mL) and washed with H_2_O(100 mL), 1 N aq.HCl solution (100 mL) sat.aq. NaHCO_3_ (2 x 100 mL) 1 N aq.HCl solution (2 x 100 mL) and brine (100 mL). The resulting CH_2_Cl_2_ layer was dried over anhydrous sodium sulfate, filtered and applied to a column of silicagel 60 (70-230 mesh, 100 x 45 mm) and eluted using a gradient elution method of CH_2_Cl_2_ : EtOAc (0 – 20%). Fractions containing the second major product to elute from the column were combined and evaporated to give an off-white foam (2.52 g, 34%). ^1^H- NMR (300 MHz, CDCl_3_) δ: 8.37 (s,0.5H); 8.26 (d, 0.5H); 8.11 (d, 0.5H); 7.97 (d, 0.5H); 7.64 (s, 0.5H); 7.08 (s, 2H); 6.75 (s, 2H); 5.56 (dd, 1H); 5.49 (d, 1H); 5.13 (dd, 1H); 5.02 (m, 1H); 4.31 (td, 1H); 4.22 (dd, 1H); 4.13 (m, 2H); 3.58 (m, 1H); 3.50 (m, 1H); 2.57 (m, 2H); 2.31 (s, 3H); 2.28 (s, 3H); 2.19 (s, 3H); 2.18 (s, 3H); 2.14 (s, 3H); 2.12 (s, 3H); 2.15 (d, 3H); 2.03 (d, 3H); 2.01 (s, 6H).

Synthesis of 5(6)-(2-dimethylaminoethyl)carboxamido)-2’7’-dichlorofluorescein-3’,6’-di-O-β-D-galactopyranoside (1).

To a flame-dried flask under anhydrous N_2_(g) conditions was added 5(6)-(2-dimethylaminoethyl)carboxamido)-2’7’-dichlorofluorescein-3’,6’-di-O-β-D-galactopyranoside, octaacetate (1.50 g, 3.0 mmol). The sample was suspended in anhydrous MeOH (100 mL), cooled to 0^o^C (ice-bath) under an atmosphere of dry N_2_(g) and 25% (w/v) sodium methoxide/MeOH solution (1.0 mL, 250 mg) was added with stirring. The solution was allowed to react under anhydrous conditions for 3 hours at 0^o^C, warmed to room temperature and neutralized with washed, dry IRC50 (H+) resin (2 grams). The resin was filtered and washed with MeOH, and the combined filtrates evaporated to dryness and dried *in vacuo* to an off white solid (1.05 grams, 98%). Crystallization from MeOH:diethyl ether (1:10, 2X) gave a product homogeneous by TLC analysis (7:3 EtOAc:MeOH irrigant; Rf = 0.08).

Synthesis of 5(6)-(2-dimethylaminoethyl)carboxamido)-2’7’-dichlorofluorescein-3’,6’-di-O-β-D-glucopyranoside. (3)

Syntheses of the β-glucosidase substrate from its peracetate *(****4****)* were performed in a similar manner to that for the β-galactoside substrates above, except using acetobromoglucose in the initial coupling step. Analysis: TLC: (irrigant = 9:1 CH_2_Cl_2_:MeOH) Rf = 0.37, 0.32; ^1^H-NMR (CDCl_3_) δ: 9.15 (s, 1H, N-H); 8.78 (s, 0.5H); 8.58 (d, 0.5H); 8.48 (d, 0.5H); 8.14 (d, 0.5H); 7.89 (s, 0.5H); 7.05 (s, 2H); 6.78 (s, 2H); 5.33 (m, 2H); 5.20 (t, 2H); 5.05 (d, 2H); 4.29 (br s, 4H); 3.93 (m, 4H); 3.28 (d, 2H); 2.90 (d, 6H); 2.15 (s, 6H); 2.05 (m, 18H).

Synthesis of 5(6)-(2-dimethylaminoethyl)carboxamido)-2’7’-dichlorofluorescein diacetate.(5)

A suspension of 5(6)-(2-dimethylaminoethyl)carboxamido)-2’7’-dichlorofluorescein (101 mg, 0.20 mmole) in anhydrous CH_2_Cl_2_ (20 mL) was cooled to 0^o^C (ice-bath) and acetic anhydride (1.0 mL, 10.6 mole) and dry pyridine (1.0 mL, 12.4 mmole) were added. This mixture was allowed to stir at 0^o^C for two hours and at ambient temperature overnight. The reaction mixture was diluted with CH_2_Cl_2_ (100 mL), poured into ice-water (100 mL), the organic layer separated and washed with ice-cold saturated sodium bicarbonate solution (1 x 100 mL), H_2_O (1 x 100 mL) and brine solution (1 x 200 mL). The resulting organic layer was dried over anhydrous sodium sulfate, filtered, evaporated and dried *in vacuo* to give a pale tan solid that was triturated with diethyl ether (30 mL), centrifuged, the supernatant ether decanted and the resulting off-white crystals dried *in vacuo* (40 mg, 33%). TLC analysis (9:1 CH_2_Cl_2_:MeOH) showed two isomers at Rf = 0.58 and 0.42) ^1^H- NMR (300 MHz, CDCl_3_) δ 8.52 (s, 0.5 H), 8.31 (d, *J* = 8.8 Hz, 0.5 H), 8.27 (s, 0.5 H), 8.19-8.12 (m, 1H), 8.00 (d, *J* = 7.4 Hz, 0.5 H), 7.89 (d, *J* = 7.4 Hz, 0.5 H), 7.40 (s, 0.5 H), 7.20 (m, 2H), 6.88 (m, 2H), 3.92 (t, *J* = 5.2 Hz, 1H), 3.74 (q, *J* = 5.2 Hz, 2H), 3.65 (m, 1H), 2.79 (br s, 6H), 2.37 (s, 6H).

Synthesis of 5(6)-(3-N-morpholinopropyl)carboxamido)-2’7’-dichlorofluorescein

O-trifluoroacetyl-N-hydroxysuccinimide (11.18 g, 53 mmol) was dissolved in anhydrous DMF (20 mL) and 5(6)-carboxy-2’,7’-dichlorofluorescein (5.20 g, 12.0 mmole) added with stirring. This mixture was allowed to stir at room temperature under anhydrous condition for 12 hours after which time, TLC analysis (mini-workup with EtOAc:H_2_O; EtOAc layer; irrigant = 7:3 EtOAc:H_2_O) indicated that the reaction was complete. The reaction mixture was poured into ice-water (300 mL) with stirring and washed with EtOAc (200 mL). The layers were separated and the aqueous layer washed again with fresh EtOAc (100 mL). The combined EtOAc layers were washed with H_2_O (200 mL), brine solution (200 mL), 1 N HCl/H_2_O (200 mL) and brine (100 mL). A significant amount of orange precipitate formed which was filtered and dried (1.79 g, appears to be one isomer, by TLC analysis (7:3 EtOAc:MeOH; Rf = 0.53). The filtrate was dried over anhydrous sodium sulfate, filtered, evaporated and dried *in vacuo* to give a bright orange solid (TLC analysis (7:3 EtOAc:MeOH irrigant), 2 isomers, Rf = 0.53 and 0.79) (4.65g, 6.44g total yield 70%). A sample of the mixed isomeric NHS esters (1.00 g, 2.17 mmole) was dissolved in anhydrous DMF, and 3-aminopropyl-N-morpholine (1.3 mL, 8.90 mmole, 4.1 equiv.) added with stirring. This mixture was allowed to stir under anhydrous conditions overnight, evaporated under vacuum to a bright red oil (1.53 g) that was triturated with EtOAc (50 mL) (30 min.) and diethyl ether (50 mL) (overnight) to remove excess morpholino compound. The ether was filtered and the precipitate redissolved in dry MeOH, evaporated and dried overnight *in vacuo* to give a red solid (542 mg, 44%) homogeneous by TLC analysis (irrigant = 7:3 EtOAc:MeOH) Rf = 0.06).

Synthesis of 5(6)-(3-N-propylmorpholinocarboxamido)-2',7'dichlorofluorescein, diacetate (6)

The sample of 5(6)-(3-N-morpholinopropyl)carboxamido)-2’7’-dichlorofluorescein (542 mg, 0.95 mmole) was dissolved in anhydrous CH_2_Cl_2_ (10 mL) and acetic anhydride (1.0 mL, 10.6 mmole) and dry pyridine (1.0 mL, 12.4 mmole) added. This reaction mixture was allowed to stir overnight under anhydrous conditions, diluted with CH_2_Cl_2_ (50 mL) and poured into ice-water (150 mL) with stirring. After stirring for 30 min. to destroy excess acetic anhydride, the layers were separated and the organic layer was washed with ice-cold saturated sodium bicarbonate solution (25 mL), 1 N HCl solution (25 mL) and H_2_O (25 mL). The organic layer was dried over anhydrous sodium sulfate, evaporated and dried *in vacuo* to a clear, pale tan oil, homogeneous by TLC analysis (irrigant = 9:1 CH_2_Cl_2_:MeOH; Rf = 0.58 and 0.55) as two closely separated isomers, quenching at UV 254 nm, but non-fluorescent. ^1^H-NMR (CDCl_3_) δ: 8.60 (m, 1.5H), 8.37 (d, *J* = 7.7 Hz, 0.5 H), 8.30 (d, *J­* = 7.7 Hz, 0.5 H), 8.15 (d, *J* = 7.7 Hz, 0.5 H), 7.69 (s, 0.5 H), 7.30 (d, *J* = 7.7 Hz, 0.5 H), 7.17 (s, 2H), 6.86 (d, *J* = 2.4 Hz, 2H), 3.98 (br s, 2H), 3.75-3.52 (m, 4H), 2.96-2.67 (m, 4H), 2.36 (s, 6H), 2.14-2.03 (m, 2H), 2,02-1.87 (m, 2H). A biocompatible staining solution was prepared by dissolving 86 mg of the above compound in anhydrous DMSO (1.312 mL) to give a 100 mM solution for use in cell analysis.

Synthesis of 4-Chloro-2-nitrosoresorcinol

A solution of anhydrous ethanol (100 mL) under dry nitrogen gas was cooled in an ice-bath (0^o^C) and solid sodium metal (2.3 g, 100 mmole) added with stirring until dissolved. 4-chlororesorcinol (14.4g, 100 mmole) was added with stirring until dissolved (20 min.) and a solution of N-butylnitrite (10.3 g, 100 mmole) in absolute ethanol (10 mL) added dropwise with stirring. This solution was allowed to react for 3 hours at 0^o^C, and then poured into ice-water (300 mL) and acidified with 1 N aqueous HCl solution (100 mL) until the pH was 3. The resulting solid was collected by filtration, washed with water and dried *in vacuo* to give a solid (6.4 g, 37%). TLC analysis showed a single spot (irrigant = 5:1 EtOAc:MeOH) Rf = 0.40.

Synthesis of 3-Chloro-5-carboxyresorufin

4-Chloro-2-nitrosoresorcinol (6.10 g, 35.1 mmole) and 3,5-dihydroxybenzoic acid (5.41 g, 3.51 mmole) was suspended in anhydrous MeOH (140 mL) and cooled to -5^o^C in an ice-MeOH bath. Solid manganese dioxide (3.40 g) was added followed by conc. sulfuric acid (3.7 mL) dropwise keeping the temperature between 0^o^ and 5^o^C. The ice-MeOH bath was removed and the dark red mixture allowed to stir at room temperature for 2 hours. The solution was then filtered through a fluted filter paper, and conc. ammonium hydroxide added until the filtrate changed to a dark green-blue color. This solution was again filtered through a Celite^TM^ 545 pad and additional ammonium hydroxide added (20 mL) to completely dissolve the sample. This basic solution was cooled in an ice-bath with stirring and the pH was adjusted to 2 with aqueous 4 N HCl solution. The resulting solution was evaporated to dryness and redissolved in MeOH (30 mL) and applied to silicagel 60, with evaporation. This solid sample was applied to a column of silicagel 60 (70-230 mesh, 45 x 2 cm) and eluted by gradient elution with 20%, 25% and 30% MeOH in CH_2_Cl_2_ (1 L each) and 40% MeOH in CH_2_Cl_2_ (500 mL). The third set of fractions contained the title dye (2.70 g, 26%). TLC (irrigant = 1:1 CH_2_Cl_2_:MeOH, Rf = 0.58).

Synthesis of 3-Chloro-5-carboxyresorufin, ethyl ester

Absolute ethanol (50 mL) was added to a dry flask under nitrogen gas and cooled in an ice-bath. Acetyl chloride (3.56 mL, to make 1 M HCl/EtOH) was added and this solution allowed to stir at 0^o^C for 15 min. A sample of 3-Chloro-5-carboxyresorufin (303 mg, 1.04 mmole) was added and the solution allowed to stir under anhydrous conditions at room temperature overnight. The solvents were evaporated at reduced pressure (rotovap) and co-evaporated with abs. ethanol (2 x 10 mL) and dried *in vacuo* to give a dark red solid (0.36 grams) that was purified by column chromatography (silicagel 60, 70-230 mesh column 25 x 400 mm, elution with 20:1 CH_2_Cl_2_:MeOH). Fractions containing the pure ethyl ester were combined and evaporated to give a red solid (41 mg, 12%). TLC analysis (9:1 CH_2_Cl_2_:MeOH) Rf = 0.39.

Synthesis of 2-chloro-6-carboxyresorufin

To a flame-dried 250 mL round-bottom flask was added 4-chloro-6-nitrosoresorcinol (6.94 g, 40.0 mmol), 2,6-dihydrobenzoic acid (6.16 g, 40.0 mmol) and sulfuric acid (40.0 mL). The solution was heated to 107 °C and stirred for 35 min. The solution was then cooled to room temperature and poured into ice-cold H_2_O (300 mL). After 15 min, the resulting red precipitate was filtered through a Buchner funnel, washing with H_2_O until a neutral pH was obtained. The precipitate was dried *in vacuo* to give the chlorocarboxyresorufin (9.48 g, 32.5 mmol, 81%) as a dark red solid.

Synthesis of 2-chloro-6-carboxyresorufin, NHS ester

To a flame-dried 250 mL round-bottom flask was added *2-chloro-6-carboxyresorufin*  (3.25 g, 11.1 mmol) and DMF (110 mL). Diisopropylethylamine (1.89 mL, 11.1 mmol) was added to enhance the solubility of the dye. *N*-hydroxysuccinimide (2.82 g, 13.4 mmol) and pyridine (1.08 mL, 13.4 mmol) were added to the stirred solution. After 22 h, the reaction solution was diluted in EtOAc (500 mL) and washed with H_2_O (3 x 200 mL) and sat. aq. NaCl (500 mL). The dried organic solution (MgSO_4_) was concentrated and dried *in vacuo* to give the NHS ester (1.12 g, 2.88 mmol, 26%) as a dark red oil.

Synthesis of 2-chloro-6-(dimethylaminoethylcarboxamido)resorufin (11)

To a flame-dried 50 mL round-bottom flask was added 2-chloro-6-carboxyresorufin, NHS ester (430.5 mg, 1.11 mmol) and DMF (6.0 mL), followed by *N*,*N*-dimethylethylenediamine (244.6 μL, 2.22 mmol). After 95 min, the product was precipitated from solution using diethyl ether (500 mL). The precipitate was dried *in vacuo* to give the dimethylaminoethyl targeted resorufin (233.8 mg, 0.646 mmol, 58%) as a very dark purple solid. ^1^H NMR (300 MHz, DMSO-*d*_6_) δ 9.23 (t, *J* = 4.7 Hz, 1H), 7.69 (s, 1H), 7.44 (d, *J* = 8.9 Hz, 1H), 6.57 (d, *J* = 8/9 Hz, 1H), 6.18 (s, 1H), 3.59 (q, *J* = 5.5 Hz, 2H), 3.13 (t, *J* = 5.3 Hz, 2H), 2.76 (s, 6H).

Synthesis of 2-chloro-6-(dimethylaminoethylcarboxamido)resorufin β-D-glucoside tetraacetate (9)

To a flame-dried flask maintained under anhydrous conditions was added 2-chloro-6-(dimethylaminoethylcarboxamido) resorufin (151.6 mg, 0.419 mmol), MeCN (8.0 mL) and CH_2_Cl_2_ (2.0 mL), followed by, sequentially, acetobromoglucose (215.6 mg, 0.524 mmol), silver carbonate (72.6 mg, 0.263 mmol) and *sym*-collidine (55.4 μL, 0.419 mmol). The suspension was stirred in the absence of light at room temperature. After 24 h, the suspension was sonicated for 30 min, then additional acetobromoglucose (216.3 mg, 0.526 mmol), silver carbonate (72.6 mg, 0.263 mmol) and *sym*-collidine (55.4 μL, 0.419 mmol) were added. After 5 days, the dark orange/black suspension was passed through a Celite plug, washing with CH_2_Cl_2_ (50 mL), and the filtrate was concentrated *in vacuo*. The resulting residue was diluted in EtOAc (200 mL) and washed with H_2_O (200 mL). The product was extracted from the organic layer with 1.0 HCl (3 x 100 mL), and the acidic aqueous layer was washed with EtOAc (200 mL). The aqueous layer was neutralized with sat. aq. NaHCO_3_, and the product was extracted with EtOAc (4 x 100 mL). The organic layer was washed with sat. aq. NaHCO_3_ (2 x 100 mL), dried over MgSO_4_ and concentrated *in vacuo*. The resulting residue was purified via preparatory TLC, eluting with 9:1 CH_2_Cl_2_/MeOH, to give the bis-glucoside peracetate substrate (15.9 mg, 0.23 mmol, 5%) as an orange solid. ^1^H NMR (300 MHz, CDCl_3_) δ 7.83 (d, *J* = 9.1 Hz, 1H), 7.68 (s, 1H), 7.22 (d, *J* = 9.1 Hz, 1H), 6.51 (s, 1H), 5.42-5.27 (m, 2H), 5.20 (d, *J* = 7.3 Hz, 1H), 5.10 (t, *J* = 5.5 Hz, 1H), 4.48-4.21 (m, 3H), 4.02-3.60 (m, 4H), 2.17 (s, 6H), 2.11-2.02 (m, 12H).

Synthesis of 2-chloro-6-(dimethylaminoethylcarboxamido)resorufin acetate (10)

To a solution of 2-chloro-6-(dimethylaminoethylcarboxamido) resorufin (73.0 mL, 0.202 mmol) in anhydrous CH_2_Cl_2_ (10.0 mL), was added dry pyridine (1.0 mL) and acetic anhydride (1.0 mL). After 18 h, the solution was diluted with CH_2_Cl_2_ (100.0 mL), washed with sat. aq. NH_4_Cl solution (3 x 50 mL) and sat. aq. NaHCO_3_ solution (3 x 50 mL). The aqueous layer was adjusted to pH 3 with 1.0 N HCl and was re-extracted with EtOAc (3 x 50 mL). The combined organic layers were dried over anhydrous MgSO_4_ and concentrated *in vacuo.* The product was then precipitated from acetone. After centrifugation, the resulting orange precipitate was washed with additional acetone, re-centrifuged, and dried *in vacuo* to give the acetate (7.4 mg, 0.018 mmol, 9% yield) as an orange solid. ^1^H NMR (300 MHz, DMSO-*d*_6_) δ 10.40 (br s, 1H), 8.24 (s, 1H), 7.78 (s, 1H), 7.72 (d, *J* = 9.6 Hz, 1H), 6.97 (d, *J* =9.6 Hz, 1H), 4.11 (t, *J* = 7.5 Hz, 2H), 3.24 (br s, 2H), 2.80 (s, 6H), 2.45 (s, 3H), 2.41 (s, 3H).

Synthesis of 2-chloro-6-(dimethylaminoethylcarboxamido)resorufin β-D-galactoside tetraacetate (8)

To a flame-dried 25 mL round-bottom flask was added 2-chloro-6-(dimethylaminoethylcarboxamido) resorufin (100.0 mg, 0.276 mmol), MeCN (5.0 mL) and CH_2_Cl_2_ (1.0 mL), followed sequentially by addition of *sym*-collidine (36.6 μL, 0.276 mmol), silver carbonate (48.8 mg, 0.177 mmol) and acetobromogalactose (143.6 mg, 0.349 mmol). This suspension was stirred at room temperature in the absence of light under anhydrous conditions. After 24 h, additional silver carbonate (49.2 mg, 0.178 mmol) and acetobromogalactose (145.8 mg, 0.355 mmol) were added to the suspension. After 3 days, the dark orange suspension was filtered through a Celite^TM^ plug, and the solids washed with additional CH_2_Cl_2_ (50 mL). The combined filtrates were concentrated to an oil *in vacuo*, redissolved in CH_2_Cl_2_ (100 mL) and was washed with H_2_O (2 x 50 mL). The organic layer was dried over anhydrous MgSO_4_, concentrated *in vacuo* and the residue purified via flash chromatography over silica gel, using gradient elution with 0% 🡪 8% MeOH/CH_2_Cl_2_. The product was further purified via preparatory TLC, eluting with 9:1 CH_2_Cl_2_/MeOH, to give the digalactoside peracetate (7.9 mg, 0.011 mmol, 4%) as a dark orange/brown solid.
